# Causal association of serum biomarkers with oral cavity and oropharyngeal cancer: a mendelian randomization study

**DOI:** 10.1186/s12903-023-03729-x

**Published:** 2023-12-09

**Authors:** Weixing Liu, Yue Liu, Pei Li, Zhiyuan Wang, Jia Chen, Hui Liu, Jin Ye

**Affiliations:** 1 Department of Otolaryngology, Head and Neck Surgery, Third Affiliated Hospital of Sun Yat-sen University, #600 Tianhe Road, Tianhe, Guangzhou, Guangdong 510630 P.R. China; 2 Division of Pulmonary and Critical Care, Department of Internal Medicine, Third Affiliated Hospital, Sun Yat-sen University, #600 Tianhe Road, Tianhe, Guangzhou, Guangdong 510630 P.R. China

**Keywords:** Serum biomarkers, C-reactive protein, Mendelian randomization, Oral cancer, Oropharyngeal cancer

## Abstract

**Background:**

Observational epidemiological studies revealed that multiple serum biomarkers can be associated with the risk of oral and oropharyngeal cancer (OC/OPC). However, the causal relationship between them remains largely unknown. This study aimed to investigate the causal relationship between potential serum biomarkers and (OC/OPC).

**Methods:**

A two-sample Mendelian randomization (MR) approach was performed to assess the causal association of 10 serum biomarkers with the risk of OC / OPC. Summary data on OC/OPC were obtained from a GWAS meta-analysis that included 2497 cases and 2928 controls. The TwoSampleMR package in R was used to perform MR analyzes. Inverse-variance weighted (IVW), Weighted median and MR-Egger methods were used to assess causal effects.

**Results:**

Suggestive associations with increased risk of C-reactive protein (CRP) (*OR* 1.52, *95% CI* 1.14 to 2.02), using the IVW method. MR-Egger regression suggested that directional pleiotropy was unlikely to bias the result (*P* = 0.19). The findings were robust to sensitivity analyzes. The risk of OC/OPC was not associated with serum 25-hydroxyvitamin D, HDL cholesterol, LDL cholesterol, total cholesterol, triglycerides, adiponectin, leptin, HbA1C and Insulin-like growth factor 1 (IGF 1).

**Conclusions:**

This study supports that CRP was causally associated with an increased risk of oral and oropharyngeal cancer.

## Background

Head and neck cancer (HNC) is a common cancer worldwide, of which 50% are oral cavity and oropharyngeal cancer (OC/OPC) [1]. More than 60% of new cases are diagnosed as advanced and the overall survival rate in advanced OC/OPC is less than 50% [2, 3]. Although human papillomavirus, chewing of betel quid, and lifestyle such as smoking and alcohol consumption are identified as risk factors for OC/OPC, its etiology is not fully known [4]. Therefore, the identification of potential risk factors is better for understanding the pathogenesis of OC/OPC and is essential to reduce the morbidity and mortality of OC/OPC. Some potential risk factors can be reflected in serum biomarkers, such as chronic inflammation, obesity, and diabetes. Each type of serum biomarker can provide different information about the state of the disease. Inflammation is an enabling characteristic of cancer [5]. C-reactive protein (CRP) is a classic biomarker of chronic inflammation, and serum CRP was correlated with various diseases [6, 7]. Several observational epidemiological studies have found an association of CRP with the incidence of OC/OPC [8, 9]. A study reported that serum lipid levels were inversely associated with the occurrence of oral cancer [10]. Furthermore, the level of adiponectin was positively associated with the risks of squamous cell carcinoma [11].However, conventional observational studies have many drawbacks, such as unmeasured confounding and reverse causation.

Mendelian randomization (MR) is a tool designed to investigate the causal relationship between exposure and disease outcome using genetic variation as instrumental variables (IVs) [12]. MR analysis using such IVs resembles randomized clinical trials and is less susceptible to confounding and reverse causation [13]. Therefore, this study used two-sample MR to investigate the causal association between serum biomarkers and the risk of OC/OPC. We used genetic variants associated with serum biomarkers as instrumental variables to improve inference for a possible influence of serum biomarkers on the risk of developing OC/OPC.

## Methods

### Serum biomarkers for SNP selection

**Fig. 1:**
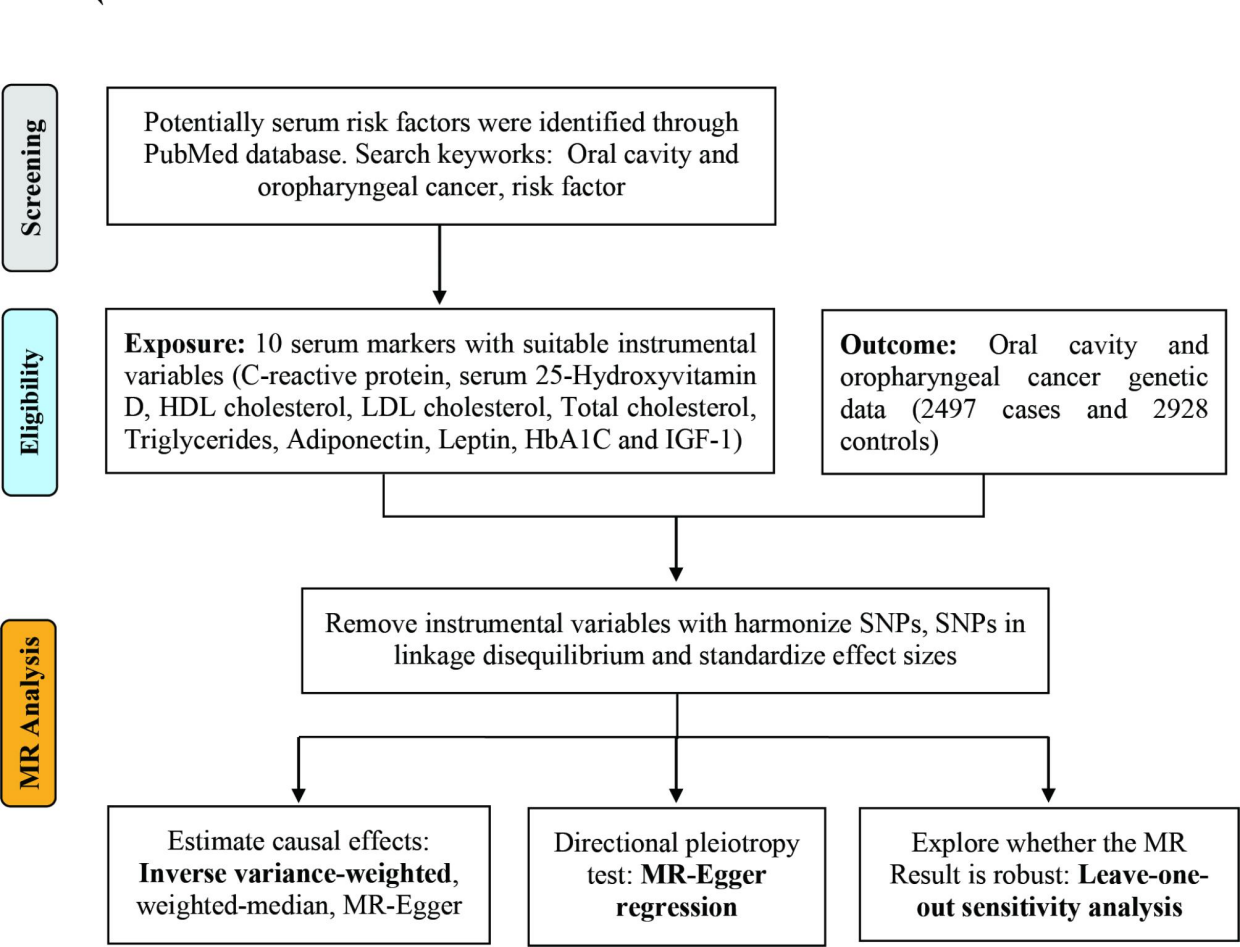
A schematic summary of the study design

Figure 1 shows a flowchart of the study design. The following search keywords were used: oral cavity and oropharyngeal cancer, risk factor. Single nucleotide polymorphisms (SNPs) associated with potential serum biomarkers were available from previously published GWAS studies of European descent. Construct genetic tools by obtaining SNPs that show robust (p < 5 × 10^−8^) and independent associations (r2 < 0.001). The data used in this study are available from previously published GWAS studies, which have relevant participant consent and ethical approval. The Ethics Committee of the Third Affiliated Hospital of Sun Yat-sen University approved this study.

### Oral cavity and oropharyngeal cancer population

A summary of European descent GWAS statistics was used for the outcome of the risk. It contains 2497 cases and 2928 controls, and the clinical characteristics of the cases and controls can be found in a previously published GWAS study [14]. The included cases met the standards of the 10th edition of the International Classification of Diseases (ICD-10). Informed consent was obtained for all participants, and the study was approved by the relevant ethics committee.

### Statistical analysis

The two-sample Mendelian randomization (MR) approach is based on three main assumptions [15]. First, genetic variants are strongly associated with serum biomarkers. Second, there are no unmeasured confounding factors related to the association of genetic variants with the outcome. Third, genetic variants affect the OC/OPC only through risk factors. Serum biomarkers with *F*-statistic < 10 was excluded to ensure the strength of the selected instruments (formula: F = R2×(N − 2)/(1 − R2), R2 = 2 × MAF × (1 − MAF) × beta2) [13, 14]. Power calculations in MR analysis were performed according to Brion et al. [16]. The inverse-variance weighted method (IVW) was used to explore the bidirectional causality between serum biomarkers and OC / OPC through a meta-analysis of SNP-specific Wald ratio estimates [13]. The causal effect estimates and equivalent beta coefficients were calculated, which were then converted into odds ratios (OR). The weighted median and MR-Egger methods were also implemented to assess causality. Many IVs were associated with multiple traits (pleiotropy). Sensitivity analysis was critical in MR studies to detect the underlying pleiotropy. Horizontal pleiotropy was performed using MR-Egger regression. When the MR-Egger intercept differs from zero (P < 0.05), it indicates horizontal pleiotropy or a violation of the MR assumption [24]. Leave-one-out sensitivity analysis was carried out by removing SNPs each time to explore whether the IVW analysis were biased by a single SNP. If there was no difference between the estimated MR result and the result after removing an IV, it indicates that the MR result is robust. The R version (4.0.3) with the package “TwoSampleMR” was used to perform MR analysis.

## Results

**Fig. 2:**
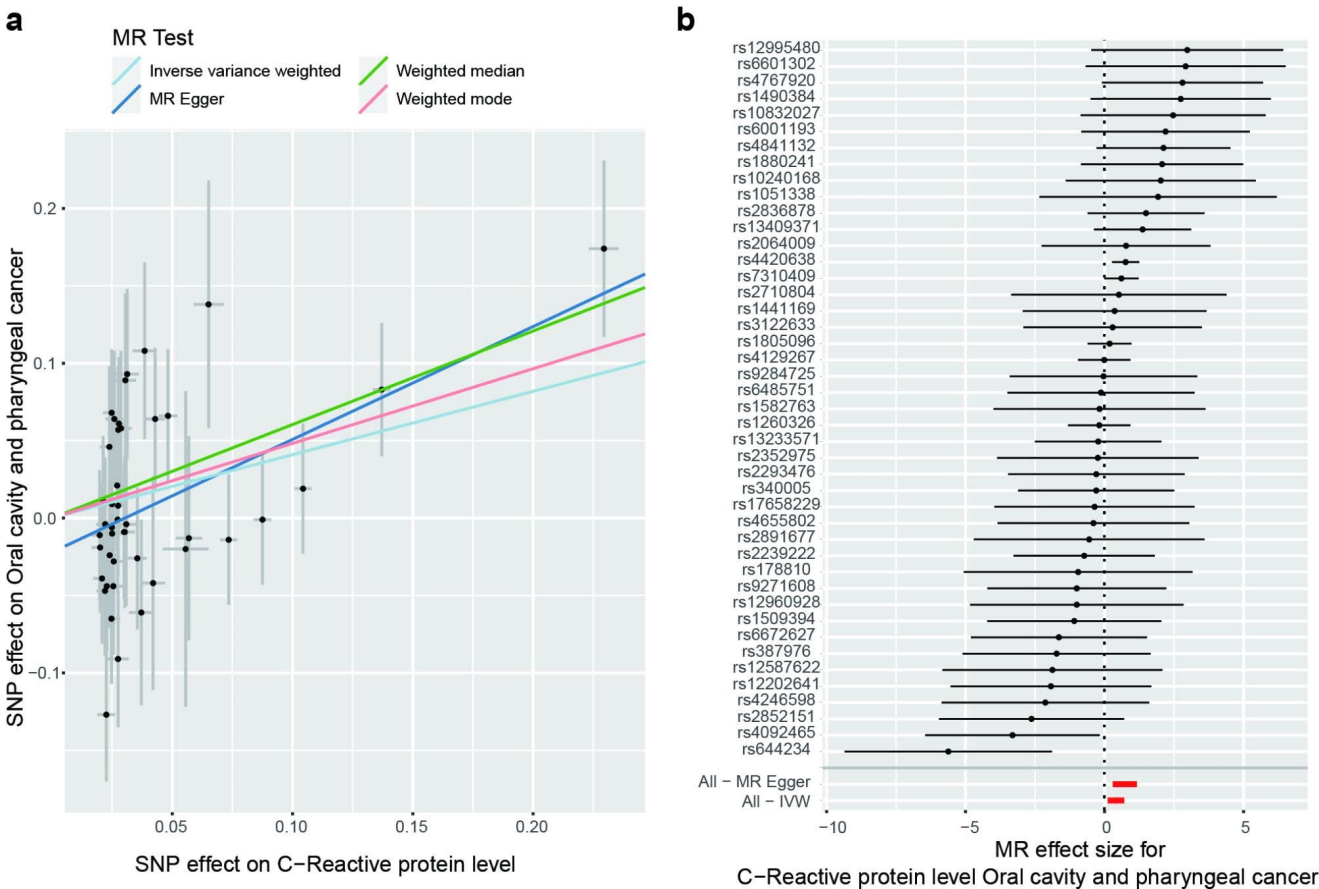
Scatter plot and Forest plot. **a** Scatter plot of the possible effects of C-reactive protein and the risk of oral cavity and pharyngeal cancer, with the slope of each line corresponding to the estimated MR effect per method; **b** Forest plot of the MR-based effect sizes of C-reactive protein exposure instruments on oral cavity and pharyngeal cancer. The horizontal red line represents the result of the MR-egger or IVW method. *Note*: *SNP* single-nucleotide polymorphism; *IVW* inverse variance weighted method

**Fig. 3:**
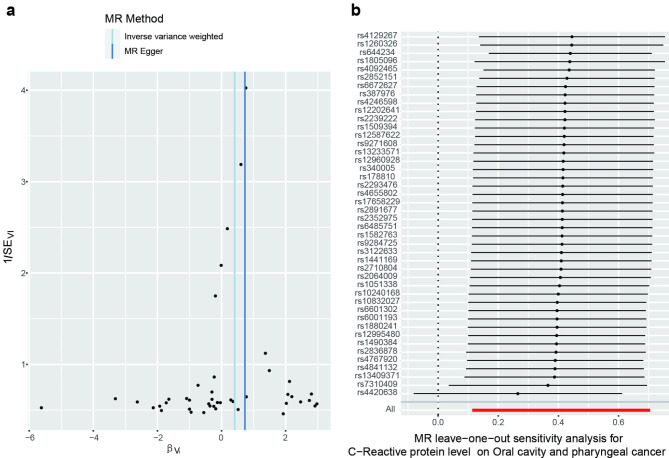
Funnel plot and Leave-one-out plot. a: Funnel plot of SNPs associated with C-reactive protein and their risk of oral cavity and pharyngeal cancer. b: Leave-one-out of SNPs associated with C-reactive protein and their risk of oral cavity and pharyngeal cancer. The horizontal red line represents the result of all SNPs using the IVW method

Various serum biomarkers have been associated with the risk of OC/OPC in observational studies, which are susceptible to confounding and reverse causation. MR analysis can overcome these limitations by using SNPs as an instrumental variable for assumed risk factors. This study investigated 10 potential serum biomarkers for the risk of OC / OPC, including C-reactive protein (CRP) [17], serum 25-Hydroxyvitamin D [18], HDL cholesterol [19], LDL cholesterol [19], Total cholesterol [19], Triglycerides [19], Adiponectin [20], Leptin [21], HbA1C [22] and insulin-like growth factor 1 (IGF-1) [23]. The sample size, the number of SNPs, the proportion of variance explained (R^2^) by the SNPs, and the F statistics for the instrument are shown in Table 1. There was a statistically significant association between CRP and OC/OPC risk (OR 1.52, 95% CI 1.14 to 2.02) using the IVW method (Table 2). Additionally, the results using the weighted median method were statistically significant (OR 1.84, 95% CI 1.24 to 2.71). Similar results of risk estimation were obtained using the MR-Egger method (OR 2.07, 95% CI 1.36 to 3.16). This study provided 98% power to detect the causal effect of CRP (OR = 1.52). Figure 2 illustrates the scatter plot and the forest plot. The *P*-values for the CRP heterogeneity tests using the MR-Egger and IVW methods were 0.19 and 0.12, respectively, indicating no heterogeneity. Furthermore, there was no significant intercept (intercept = -0.02; SE = 0.01, *P* = 0.06), which indicated no horizontal pleiotropy. The CRP funnel plot was symmetrical, indicating no pleiotropic effects (Fig. 3a). In the leave-one-out sensitivity analysis, regardless of which SNP was removed, there was no fundamental effect on OC / OPC, which means that the MR result was robust (Fig. 3b).

**Table 1:** 10 serum biomarkers for oral cavity and oropharyngeal cancer included in Mendelian randomization analysis

Reported risk factor	Sample size	nSNPs	R^2^	F statistic
C-Reactive protein level [17]	204,402	46	0.069	15,148.872
Serum 25-Hydroxyvitamin D [18]	417,580	84	0.044	19,219.071
HDL cholesterol [19]	94,595	64	0.081	8337.3591
LDL cholesterol [19]	94,595	53	0.139	15,271.111
Total cholesterol [19]	94,595	57	0.088	9127.3947
Triglycerides [19]	94,595	41	0.048	4769.395
Adiponectin [20]	39,883	9	0.048	2010.8067
Leptin [21]	27,947	5	0.010	277.92911
HbA1c [22]	46,368	8	0.048	2337.7815
IGF-I [23]	358,072	188	0.094	37,150.751

**Table 2:** Inverse-variance weighted (IVW), weighted median, and MR-Egger analysis estimates for the association of 10 serum biomarkers with oral cavity and oropharyngeal cancer risk

Risk factor	nSNPS	Inverse variance weighted		MR-Egger		Weighted median	
		*OR* *95%CI*	*P*	*OR* *95%CI*	*P*	*OR* *95%CI*	*P*
C-Reactive protein level	46	1.52 (1.14–2.02)	*P* < 0.01	2.07 (1.36–3.16)	*P* < 0.01	1.84 (1.24–2.71)	*P* < 0.01
Serum 25-Hydroxyvitamin D levels	84	1.40 (0.86–2.29)	0.18	1.02 (0.44–2.36)	1	1.05 (0.46–2.40)	0.91
HDL cholesterol	64	1.00 (0.78–1.30)	0.97	1.23 (0.83–1.83)	0.3	1.13 (0.80–1.60)	0.48
LDL cholesterol	53	1.09 (0.82–1.46)	0.54	0.98 (0.56–1.70)	0.9	1.17 (0.78–1.75)	0.45
Total Cholesterol	57	0.83 (0.60–1.13)	0.24	1.05 (0.53–2.10)	0.9	0.95 (0.60–1.51)	0.83
Triglycerides	41	0.86 (0.62–1.20)	0.37	0.94 (0.55–1.60)	0.8	0.88 (0.58–1.33)	0.55
Adiponectin	9	0.91 (0.50–1.64)	0.75	0.56 (0.2–1.58)	0.3	0.78 (0.38–1.58)	0.49
Leptin	5	1.46 (0.81–2.61)	0.21	1.94 (0.03-122.81)	0.8	1.28 (0.61–2.68)	0.52
HbA1C	8	0.83 (0.33–2.09)	0.69	0.39 (0.05–2.98)	0.4	0.51 (0.15–1.70)	0.27
IGF-I	188	1.06 (0.79–1.42)	0.69	1.23 (0.65–2.31)	0.5	1.18 (0.78–1.78)	0.42

No association was predicted between serum 25-hydroxyvitamin D, HDL cholesterol, LDL cholesterol, total cholesterol, Triglycerides, Adiponectin, Leptin, HbA1C and IGF-1, and the risk of OC/OPC was predicted (Table 2).

## Discussion

In the current study, a two-sample MR method was used to explore the correlation between 10 serum biomarkers and OC/OPC in a European population. MR analysis revealed that serum CRP levels were associated with an increased risk of OC/OPC, which may provide some of the strongest evidence to assess the causal role of CRP in OC/OPC. This study indicated an inflammatory mechanism for development and could have clinical utility in the identification of high-risk individuals. These findings reflect a recently published MR study of colorectal, breast, and gallbladder that supports the causal role of CRP in the risk of these cancers [24–26].

Chronic inflammation leads to cell hyperproliferation, activating a variety of cell functions, leading to malignant changes in DNA [27]. Inflammatory mediators such as nuclear factor-kappa (NF-κB) and cytokines are the main mediators involved in the pathogenesis of cancer [28]. NF-κB plays an important role in innate immunity/inflammation. Moreover, NF-κB is recognized as a crucial player in the initiation and progression [29]. CRP could be a biomarker of inflammation in the tumor microenvironment (TME), and chronic inflammation plays an active role in cell proliferation and tumorigenesis [30]. Chronic inflammation can contribute to epithelial cell mutations and epigenetic changes and can also serve as direct growth factors for tumor growth [31]. CRP could potentially promote CEA, MMP1 and MMP2 by stimulating LOX-1 receptors in colorectal cancer [32]. Chronic inflammation in the oral cavity was associated with an increased risk of HNC [33, 34]. Furthermore, smoking and heavy drinking can also cause chronic inflammation of the oral mucosa. Serum CRP level was significantly associated with the prognosis of HNC patients, and can be recommended for prognostic evaluation in clinical work [35]. Furthermore, high levels of CRP can predict lymph node metastasis, advanced tumor stage, and recurrence in OC [36]. Mechanically, CRP has the potential to solve the inflammatory environment by directly binding fibronectin to modulate fibronectin-mediated monocyte adhesion [37]. Fibronectin can regulate cell adhesion and signaling to promote oral cancer cell migration [38]. Similarly, it has the ability to stimulate white blood cells to produce IL-8 [39]. Previous studies have shown that the level of IL8 was associated with a worse overall survival in HNC patients [40]. Furthermore, a recent study found that consistent long-term use of non-steroidal anti-inflammatory drugs can be associated with a reduced risk of HNC [41]. One researcher found that anti-inflammatory drugs can reduce serum CRP [42]. Therefore, anti-inflammatory drugs can reduce the risk of HNC. Furthermore, future research could delve deeper into the specifics of these drugs and how they interact with CRP and impact the risk of OC / OPC. Taking into account this diversity of physiological activities, we can better understand the relationship between inflammation and HNC. In the future, more studies are needed to focus on the mechanism of the relationship between chronic inflammation and HNC. In addition, serum CRP should be used in OC/OPC screening tests, and patients with chronic high CRP levels need further oral and oropharyngeal examination for early detection of OC/OPC. Furthermore, the use of CRP in patient assessment can improve clinical evaluation of HNC progression.

The strength of this analysis was that an MR method was used to assess the causal relationship between CRP and OC/OPC risk, which can reduce the confounding and reverse causal bias inherent in observational studies. This MR study had sufficient power to detect moderate correlation and was unlikely to be affected by weak instrument bias. This study also has some limitations. First, this study only evaluated European ancestry to reduce the risk of population stratification, which was a well-known source of confounders in genomic data [43]. However, consistent associations have been demonstrated between CRP and oral cancer risk in Asian population [8]. The study by Zhu et al. indicated that CRP was a biomarker to assess cancer risks, which included the European, Asian, and African population [44]. Second, HPV data were not available in these summary results. HPV may contribute to the development of chronic inflammation [45, 46]. HPV-positive OC/OPC are unique from other OC/OPC and the incidence of HPV-positive OC/OPC has increased in recent years [47]. A prospective cohort study has reported that serum CRP was higher in HPV-positive than HPV-negative OPC [9]. Thus, HPV may influence the association between CRP and OC/OPC. In the future, a broader range of studies should be considered, including the OC/OPC subgroup, diverse populations, and the positive / negative HPV subgroup. Furthermore, future studies should also look at other potential biomarkers in addition to CRP that might correlate with OC/OPC.

## Conclusion

In summary, this study supports a causal relationship between CRP levels and an increased risk of oral and oropharyngeal cancers.

## Data Availability

The data sets used during the current study are available from the corresponding author on reasonable request.

## List of abbreviations

CRP: C-reactive protein
HNC: Head and Neck cancer
IV: instrumental variable
IVW: inverse variance weighted
MB: mega base
MR: Mendelian randomization
OC/OPC: oral cavity and oropharyngeal cancer
OR: odds ratio
SNP: single nucleotide polymorphism
